# FOXD1 facilitates pancreatic cancer cell proliferation, invasion, and metastasis by regulating GLUT1-mediated aerobic glycolysis

**DOI:** 10.1038/s41419-022-05213-w

**Published:** 2022-09-03

**Authors:** Kun Cai, Shiyu Chen, Changhao Zhu, Lin Li, Chao Yu, Zhiwei He, Chengyi Sun

**Affiliations:** 1 Department of Hepatic-Biliary-Pancreatic Surgery, The Affiliated Hospital of Guizhou Medical University, Guiyang, 550001 China; 2 College of Clinical Medicine, Guizhou Medical University, Guiyang, 550001 China; 3 Guizhou Provincial Institute of Hepatobiliary, Pancreatic and Splenic Diseases, Guiyang, 550001 China; 4 Key Laboratory of Liver, Gallbladder, Pancreas and Spleen of Guizhou Medical University, 550001 Guiyang, China; 5 College of Basic Medicine, Guizhou Medical University, 550001 Guiyang, China; 6 Department of Hepatic-Biliary-Pancreatic Surgery, The Affiliated Cancer Hospital of Guizhou Medical University, Guiyang, 550001 China; 7 Department of Hepatobiliary Surgery, Shenzhen Key Laboratory, Shenzhen University General Hospital, Shenzhen, 518000 China; 8 Shenzhen University Clinical Medical Academy Center, Shenzhen University, Shenzhen, 518000 China

**Keywords:** Pancreatic cancer, Prognostic markers

## Abstract

Although FOXD1 has been found to be involved in the malignant processes of several types of cancers, its role in pancreatic cancer (PC) is not well understood. This study aimed to investigate the expression and function of FOXD1 in PC. We found that FOXD1 mRNA and protein expression were upregulated in PC tissues compared with non-tumor tissues, and high expression level of FOXD1 was associated with an adverse prognostic index of PC. The results of in vitro and in vivo assays indicate that overexpression of FOXD1 promotes aerobic glycolysis and the capacity of PC cells to proliferate, invade, and metastasize, whereas FOXD1 knockdown inhibits these functions. The results of mechanistic experiments suggest that FOXD1 can not only directly promote SLC2A1 transcription but also inhibit the degradation of SLC2A1 through the RNA-induced silencing complex. As a result, FOXD1 enhances GLUT1 expression and ultimately facilitates PC cell proliferation, invasion, and metastasis by regulating aerobic glycolysis. Taken together, FOXD1 is suggested to be a potential therapeutic target for PC.

## Introduction

Pancreatic cancer (PC) is the most lethal disease of the digestive system [1]. Because of the lack of typical clinical symptoms and effective screening methods, <20% of patients with PC are diagnosed in the early stages of the disease [2]. Radical resection is not possible in the majority of patients, and although indications for resection have been extended and attempts have been made to optimize adjuvant therapy in the past few decades, the 5-year survival rate is still below 10% [3], and the incidence and mortality rates of PC are increasing annually worldwide [4, 5]. Therefore, there is an urgent need to elucidate the pathogenesis and mechanism of disease progression to develop effective therapeutic targets for PC.

Forkhead box D1 (FOXD1) is a member of the evolutionarily conserved Forkhead box (FOX) family of genes [6, 7]. Numerous studies have shown that FOXD1 performs multiple roles in normal physiological function as well as disease progression, as is the case in kidney development [8], osteoarthritis [9], recurrent pregnancy loss [10], and malignant biological functions of various tumors [11–15]. However, the expression and biological function of FOXD1 in PC are largely unknown.

In recent years, with increased focus on the etiology mechanism of tumors, there is strong evidence that carcinogenesis, disease progression, and prognosis of PC are significantly affected by reprogrammed metabolism [16, 17]. Aerobic glycolysis, also called the Warburg effect, is a typical metabolic rearrangement in PC cells, which provides abundant ATP and minimum reactive oxygen species during the process of proliferation, migration, and invasion of PC cells [18].

Solute carrier family 2 member 1 (SLC2A1), which encodes the protein of a crucial glucose transporter, GLUT1, is overexpressed in diverse cancers, including PC, and correlated with poor clinical outcomes [19, 20]. Targeting SLC2A1 or GLUT1 has been shown to result in significant inhibition of tumor effects in both cell and animal studies of multiple tumors [20–23]. Accordingly, altering the expression or adjusting the function of SLC2A1 or GLUT1 may be a feasible strategy for treating PC.

In the present study, we found that FOXD1 is upregulated in PC and closely related to unfavorable clinical outcomes. Further investigations revealed that FOXD1 binds to the promoter and actives the transcription of SLC2A1 and lncRNA HOXA11-AS and that HOXA11-AS acts as a competing endogenous RNA (ceRNA) for miR-148b-3p, leading to increased stability of the SLC2A1 mRNA. These changes increased the expression of GLUT1, enhanced the level of aerobic glycolysis, and ultimately promoted the proliferation, invasion, and metastasis of PC cells. On the basis of these results, which demonstrate the role and mechanism of FOXD1 in promoting PC progression, we propose that FOXD1 is a promising therapeutic target for PC.

## Results

### FOXD1 is overexpressed in PC tissues and cells

**Fig. 1:**
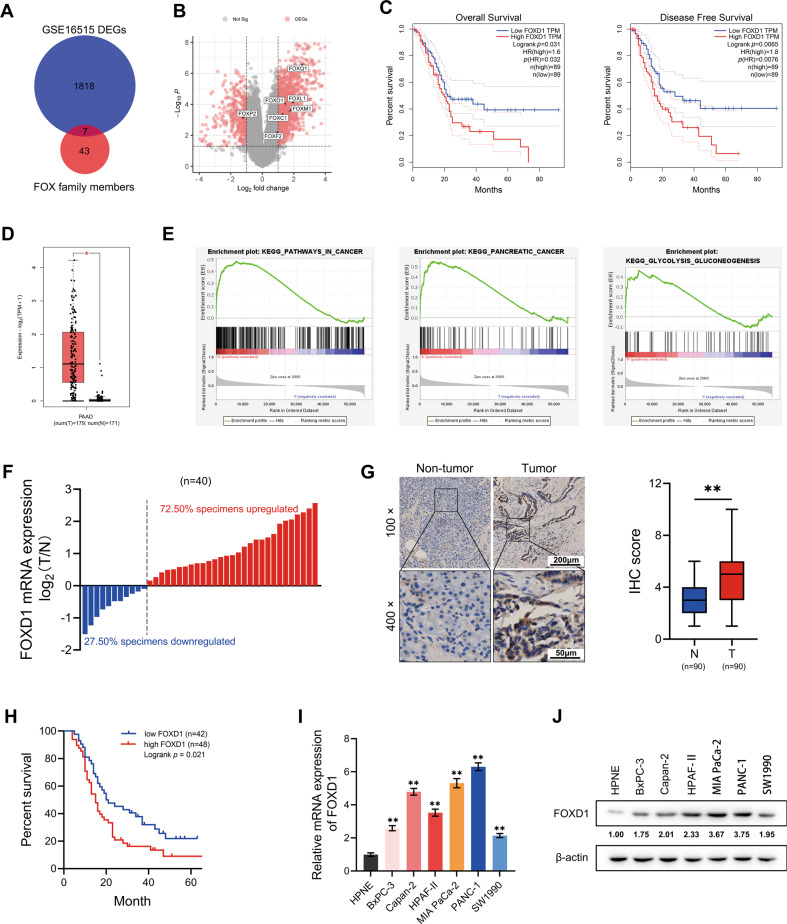
FOXD1 is overexpressed in PC tissues and cells. **A** The intersection between differentially expressed genes (DEGs) in GSE16515 and FOX family members, represented as a Venn diagram. **B** Volcano map of the DEGs in GSE16515. **C** Overall survival curve and disease-free survival curve of FOXD1 differentially expressed PC patients based on TCGA database. **D** Relative expression of FOXD1 in 179 PC(T) tissues and 171 non-tumor (N) tissues based on TCGA and GTEx database. **E** GSEA analysis of FOXD1 expression-related genes. **F** Relative FOXD1 expression in 40 pairs of PC (T) tissues and non-tumor (N) tissues detected by RT-qPCR. **G** Relative FOXD1 expression in 90 pairs PC (T) tissues and non-tumor (N) tissues detected by immunohistochemistry (IHC). **H** Overall survival curve of 90 PC patients classified by IHC score. **I** Relative FOXD1 expression in HPNE cells and PC cells detect by RT-qPCR. **J** Relative FOXD1 expression in HPNE cells and PC cells detect by western blotting analysis. *p < 0.05; **p < 0.01.

Previous studies have demonstrated that members of the FOX family of genes play different but crucial roles in the occurrence and development of multifarious tumors [6]. Here, by analyzing the GSE16515 data set, seven FOX genes (FOXC1, FOXD1, FOXF2, FOXL1, FOXM1, FOXP2 and FOXQ1) were found to be differentially expressed between PC and normal pancreatic tissues (Fig. 1A, B). Furthermore, their expression was analyzed in relation to overall survival and disease-free survival in patients with PC (ascertained with the TCGA data set). The results indicate that the high expression of FOXC1, FOXD1, FOXM1, and FOXL1 is associated with a shorter duration of both overall survival and disease-free survival in patients with PC (Fig. 1C and Supplementary Fig. S1). The expression and function of FOXD1 in PC has seldom been reported. Our analysis of the TCGA and GTXs data also showed that FOXD1 expression was higher in PC tissues than in non-tumor tissues (Fig. 1D) and that FOXD1 expression-related genes were significantly enriched in a number of pathways, namely PATHWAY_IN_CANCER, PANCREATIC_CANCER, and GLYCOLYSIS_ GLUCONEOGENESIS (Fig. 1E). We then measured the expression level of FOXD1 in the specimens. In 40 cryopreserved tissues, FOXD1 mRNA was found to be higher in 72.50% of PC(T) tissues compared with non-tumor (N) tissues (Fig. 1F). In 90 paraffin-embedded pancreatic and non-tumor tissues, FOXD1 protein expression level was higher in PC(T) tissues than in non-tumor (N) tissues (Fig. 1G). High FOXD1 expression was also found to be associated with T classification and N classification (Table 1) and shorter overall survival in PC patients (Fig. 1H). Nevertheless, compared with HPNE cells, the six PC cell lines showed high expression of FOXD1 (Fig. 1I, J).

**Table 1:** The relationship between FOXD1 expression and PC clinicopathologic features.

Clinicopathologic feature	FOXD1 expression		*p*
	Low	High	
All cases	42	48	
Age			
≤60	19	20	0.733
>60	23	28	
Gender			
Male	25	29	0.931
Female	17	19	
T classification			
T1	24	17	**0.039**
T2~T4	18	31	
N classification			
N0	30	23	**0.024**
N1/N2	12	25	
M classification			
M0	37	40	0.522
M1	5	8	
AJCC stage			
I/II	31	30	0.252
III/IV	11	18	

Bold values indicates statistically significant *p* values less than 0.05.

### FOXD1 promotes PC cell aerobic glycolysis, proliferation, invasion, and metastasis in vitro

**Fig. 2:**
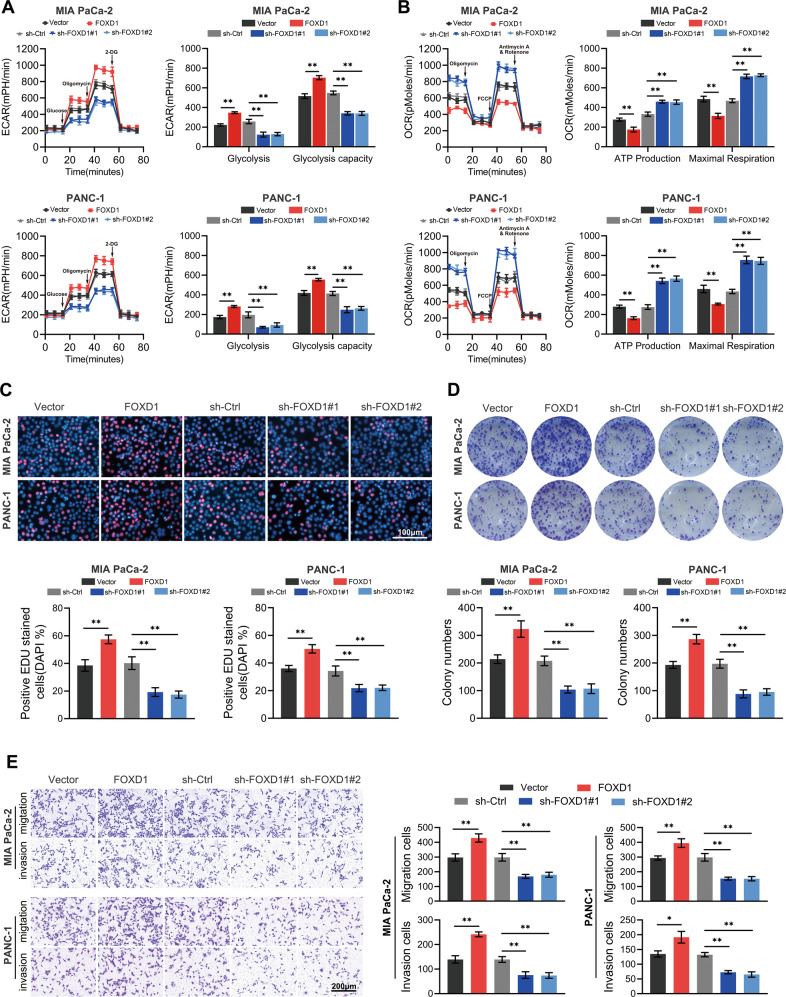
FOXD1 promotes PC cell aerobic glycolysis, proliferation, invasion and metastasis in vitro. **A** Glycolysis and glycolysis capacity of MIA PaCa-2 and PANC-1 cells with different FOXD1 expression levels were verified using ECAR. **B** ATP production and maximal respiration of MIA PaCa-2 and PANC-1 cells with different FOXD1 expression levels were verified on the basis of oxygen consumption rate (OCR). **C** Representative EdU staining images of MIA PaCa-2 and PANC-1 cells with different FOXD1 expression levels. **D** Representative colony formation images of MIA PaCa-2 and PANC-1 cells with different FOXD1 expression levels. **E** Representative Transwell images of MIA PaCa-2 and PANC-1 cells with different FOXD1 expression levels. *p < 0.05; **p < 0.01.

PC cells can shift their glucose metabolism pattern to aerobic glycolysis in order to support the vigorous capability of proliferation, invasion and metastasis. Based on the above findings, we hypothesize that FOXD1 participates in the regulation of aerobic glycolysis, thus affecting the prognosis of PC. First, stable FOXD1 overexpression, knockdown and their corresponding control MIA PaCa-2 and PANC-1 cell lines were constructed (Supplementary Fig. S2). Then we analyzed the effect of FOXD1 on glycolysis of PC cells MIA PaCa-2 and PANC-1 by using the Seahorse XF extracellular flux analyzer. The results show that upregulation of FOXD1 strengthened the glycolysis and glycolysis capacity; conversely, downregulation of FOXD1 impeded glycolysis (Fig. 2A). In addition, upregulation of FOXD1 decreased ATP production and maximal respiration. Accordingly, downregulation of FOXD1 increased ATP production and maximal respiration (Fig. 2B). The EdU staining assay and colony formation assay were then performed to confirm the role of FOXD1 in proliferation. The results showed that when PANC-1 cells and MIA PaCa-2 cells overexpressed FOXD1, the percentage of EdU-positive cells was higher and the number of clones was greater. The opposite phenomena were observed when FOXD1 expression was depressed (Fig. 2C, D). In addition, the results of the Transwell assay showed that upregulation of FOXD1 promoted—and downregulation of FOXD1 inhibited—the migration and invasion ability of PC cells (Fig. 2E). These data indicate that FOXD1 may accelerate PC progression and aerobic glycolysis in vitro.

### FOXD1 promotes PC cell proliferation and metastasis in vivo

**Fig. 3:**
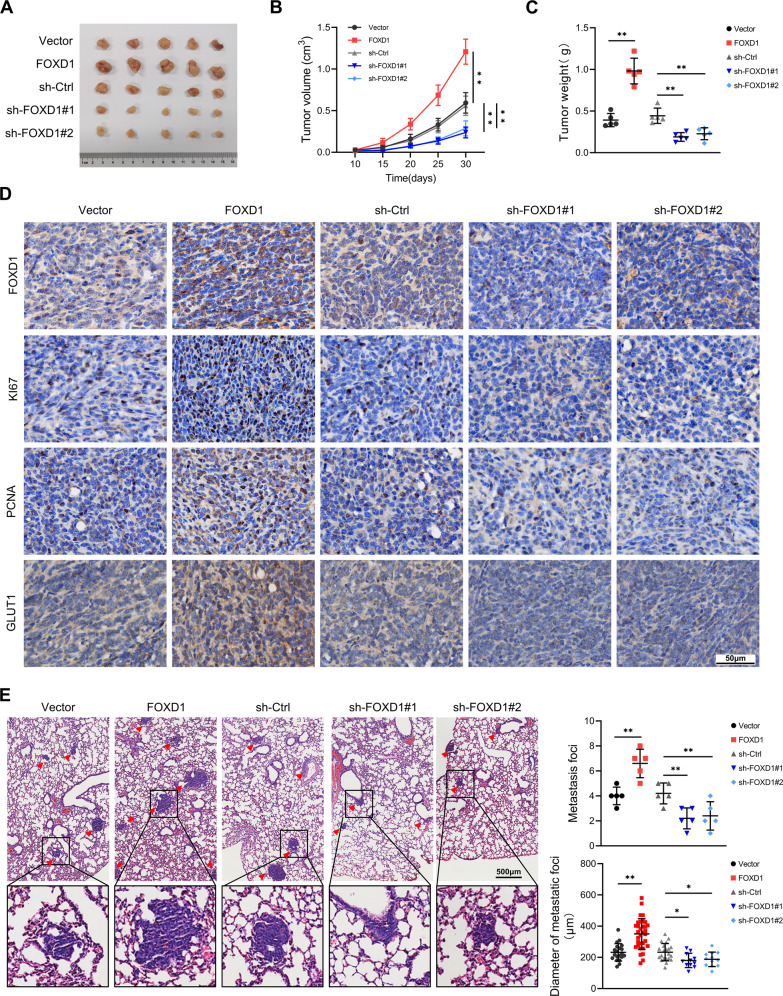
FOXD1 promotes PC cell proliferation and metastasis in vivo. **A** Excised subcutaneous tumors 30 days after inoculation with the indicated PANC-1 cells. **B** Growth curves of subcutaneous tumor volume in each group. **C** Weight of subcutaneous tumors in each group. **D** Representative images of FOXD1, KI67, PCNA and GLUT1 expression in subcutaneous tumors detected through IHC. **E** Representative images of pulmonary metastases in each group. *p < 0.05; **p < 0.01.

To further clarify the role of FOXD1 in the progression of pancreatic cancer, a subcutaneous tumorigenesis model and a lung metastasis model were established in nude mice. Relatively larger-volume and heavier subcutaneous tumors were found in mice that had been injected with FOXD1-overexpressed PANC-1 cells. In contrast, relatively smaller (volume) and lighter subcutaneous tumors were found in mice injected with FOXD1-depressed PANC-1 cells (Fig. 3A–C). IHC results showed a relatively higher abundance of KI67, PCNA and GLUT1 were found when FOXD1 was upregulated and a lower abundance of KI67 PCNA, and GLUT1 when FOXD1 was downregulated (Fig. 3D). An analysis of the lung metastasis indicated that the FOXD1 overexpression group had more metastases foci and the metastases foci were relatively larger in diameter (Fig. 3E). Together, the results of these animal models suggest that FOXD1 has the ability to promote PC cells proliferation and metastasis in vivo.

### FOXD1 activates the transcription of SLC2A1 and HOXA11-AS

**Fig. 4:**
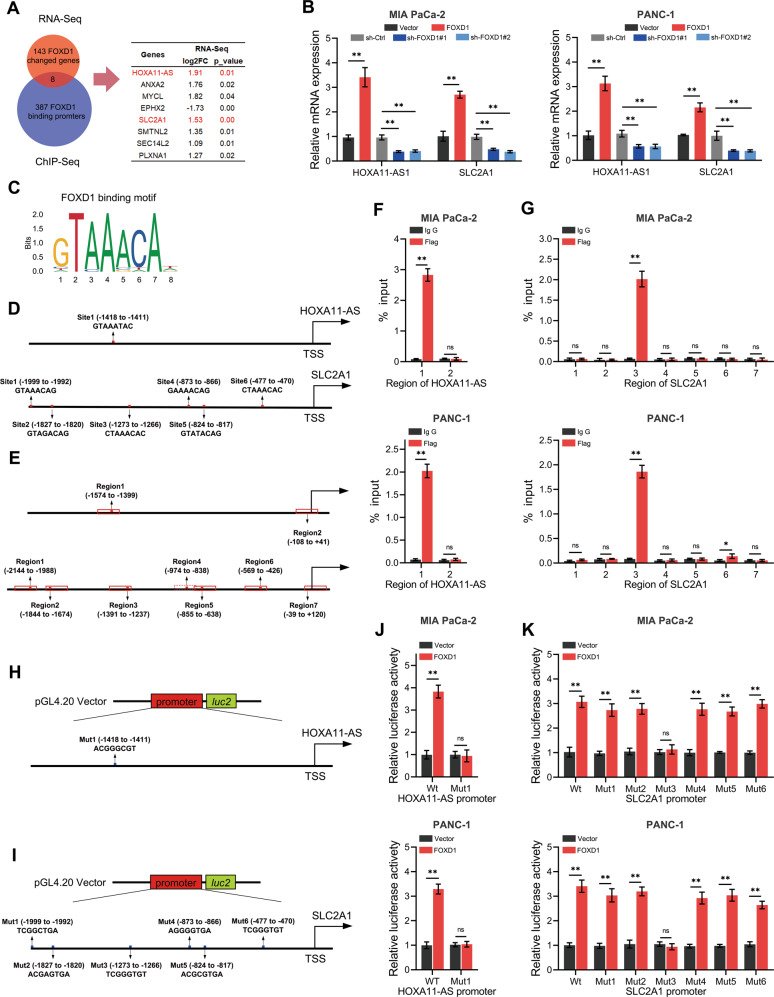
FOXD1 activates the transcription of SLC2A1 and HOXA11-AS. **A** The intersection between RNA-Seq and ChIP-Seq in FOXD1 overexpression PANC-1 cell and control cells, represented as a Venn diagram. **B** Relative HOXA11-AS and SLC2A1 expression of MIA PaCa-2 and PANC-1 cells with FOXD1 overexpression and knockdown were detected by RT-qPCR. **C** FOXD1 binding motif predicted by JASPAR. **D** Schematic diagram of potential FOXD1 binding sites in the promoter region of HOXA11-AS and SLC2A1. **E** Schematic diagram of primers designed for regions of HOXA11-AS and SLC2A1 promoter. **F**, **G** ChIP-PCR analysis of enrichment of FOXD1 on HOXA11-AS or SLC2A1 promoter. IgG was used as a negative control. **H**, **I** Schematic diagram of dual-luciferase reporter vector construction for HOXA11-AS or SLC2A1 promoter. **J**, **K** The luciferase activity of the wild-type and mutant HOXA11-As or SLC2A1 promoters with indicated cells. *p < 0.05; **p < 0.01.

Transcription factors can bind to specific gene promoter sequences and regulate the transcription of downstream genes and their subsequent function. To further investigate the genes regulated by FOXD1, RNA and ChIP sequencing were conducted. Eight of the 151 genes that changed after FOXD1 overexpression in PANC-1 cells have specific binding to Flag-tagged FOXD1 (ChIP-seq results, Fig. 4A). Notably, among these genes were *HOXA11-AS*, which is an essential initiator and facilitator in multiple cancers, and *SLC2A1*, which encodes the major glucose transporter GLUT1. The RT-qPCR results showed that overexpression of FOXD1 promoted the mRNA expression levels of HOXA11-AS and SLC2A1 in PANC-1 and MIA PaCa-2 cells, whereas knockdown of FOXD1 inhibited the mRNA expression levels (Fig. 4B). We compared the promoter region sequences of HOXA11-AS and SLC2A1 with the FOXD1 binding sequences obtained from the JASPAR database (Fig. 4C). The results showed that there could be one FOXD1 binding site on the HOXA11 promoter sequence and six FOXD1 binding sites on the SLC2A1 promoter sequence (Fig. 4D). We then designed PCR primers for the roughly 200-bp sequence that contains the possible binding sites and transcriptional start sites (Fig. 4E). The ChIP-PCR results showed that the binding sequence of the HOXA11 and SLC2A1 promoter to FOXD1 could be amplified by DNA precipitated by Flag-tagged FOXD1, which suggests that FOXD1 binds directly to the HOXA11 and SLC2A1 promoter regions (Fig. 4F, G). To further investigate the transcription activity of FOXD1, the promoter sequences containing wild-type (Wt) and each possible binding site mutant (Mut) were cloned into the pGL4.20 plasmid and transfected into FOXD1 overexpression and control PC cells (Fig. 4H, I). The luciferase assay showed that FOXD1 overexpression increased the activity of the HOXA11-AS and SLC2A1 promoter but had no effect when the FOXD1 binding site was mutant (Fig. 4J, K). Thus, FOXD1 could promote transcription of HOXA11-AS and SLC2A1 by directly binding to their promoter regions.

### HOXA11-AS upregulates SLC2A1 by sponging miR-148b-3p

**Fig. 5:**
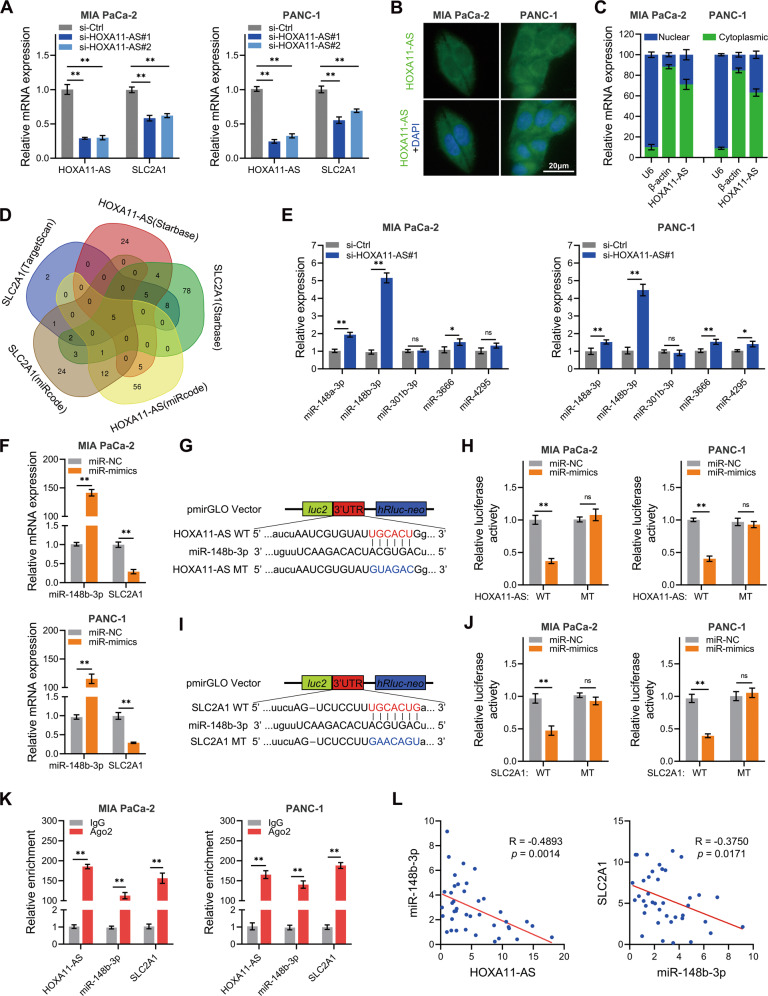
HOXA11-AS upregulates SLC2A1 by sponging miR-148b-3p. **A** HOXA11-AS and SLC2A1 were detected with RT-qPCR when HOXA11-AS was inhibited. **B**, **C** Subcellular localization of HOXA11-AS was detected by FISH or nucleocytoplasmic fractionation RT-qPCR. **D** Potential miRNAs regulated by HOXA11-AS which can bind with SLC2A1 were forecasted with indicated websites. **E** Predicted miRNAs were detected by RT-qPCR when HOXA11-AS was inhibited. **F** miR-148b-3p and SLC2A1 were detected with RT-qPCR when miR-148b-3p was overexpressed. **G** Predicted binding sites between HOXA11-AS and miR-148b-3p. **H** Luciferase activities were detected when cells co-transfected with miR-148b-3p and 3′UTR of HOXA11-AS. **I** Predicted binding sites between miR-148b-3p and SLC2A1. **J** Luciferase activities were detected when cells co-transfected with miR-148b-3p and 3′UTR of SLC2A1. **K** RIP assay was conducted to confirm the interaction between HOXA11-AS, miR-148b-3p, and SLC2A1. **L** Correlations between HOXA11-AS, miR-148b-3p, and SLC2A1 expression in 40 clinical samples was detected by RT-qPCR. *p < 0.05; **p < 0.01.

On one hand, it is interesting to note that when HOXA11-AS was inhibited in PANC-1 and MIA PaCa2 cells, the expression of SLC2A1 decreased (Fig. 5A). On the other hand, subcellular localization results showed that HOXA11-AS was mainly localized in the cytoplasm of PC cells (Fig. 5B, C). We hypothesized that HOXA11-AS functions as a ceRNA and regulates the expression of SLC2A1 in PC cells. Using TargetScan, Starbase, and miRcode (Fig. 5D), we predicted that five miRNAs, namely miR-148a-3p, miR-148b-3p, miR-301b-3p, miR-3666, and miR-4295, were possible binding miRNAs of HOXA11-AS and SLC2A1. The RT-qPCR results showed that miR-148b-3p expression did indeed increase after HOXA11-AS interference, whereas SLC2A1 expression significantly decreased after miR-148b-3p overexpression (Fig. 5E, F). The results of the dual-luciferase assay demonstrated that luciferase activity was significantly reduced when co-transfecting miR-148b-3p mimics and Wt of 3′UTR of HOXA11-AS or SLC2A1 but not in the mutant binding site type (Mut) of 3′UTR (Fig. 5G–J). The immunoprecipitation of HOXA11-AS, miR-148b-3p, and SLC2A1 by Ago2 indicated that miR-148b-3p potentially interacted with HOXA11-AS and SLC2A1 in RNA-induced silencing complex (RISC) (Fig. 5K). Furthermore, co-expression analysis indicated that the expression of miR-148b-3p was negatively correlated with HOXA11-As and SLC2A1 in 40 cases of PC tissues (Fig. 5L) and the TCGA database (Supplementary Fig. S3). Collectively, these findings suggest that HOXA11-AS upregulates SLC2A1 by sponging miR-148b-3p in PC.

### HOXA11-AS/miR-148b-3p/SLC2A1 axis and aerobic glycolysis are essential for FOXD1-mediated PC progression

**Fig. 6:**
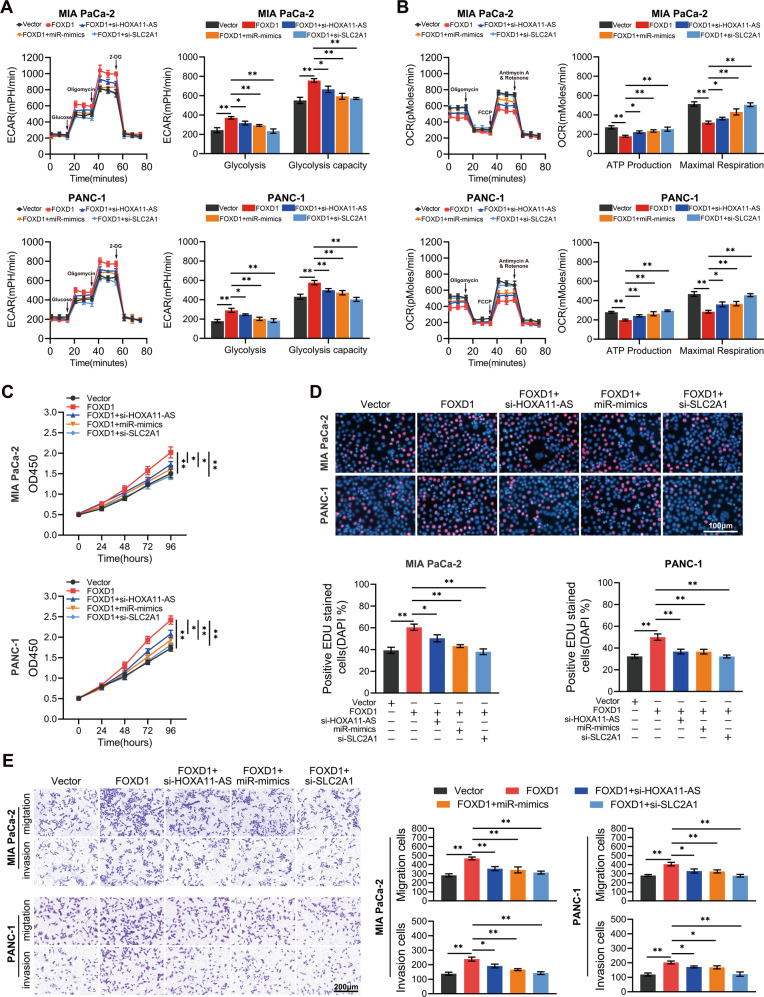
The HOXA11-AS/miR-148b-3p/SLC2A1 axis is essential for FOXD1-mediated PC progression. PC cells infected with FOXD1-overexpressing lentivirus were co-transfect with si-HOXA11-AS, miR-148b-3p mimics, and si-SLC2A1. **A**, **B** ECAR and OCR were employed to measure the level of aerobic glycolysis in each group. **C**, **D** CCK8 and EdU staining were employed to evaluate the proliferation capacity. **E** Transwell assays were performed to evaluate the invasive and metastatic capacities. *p < 0.05; **p < 0.01.

To determine whether the HOXA11-AS/miR-148b-3p/SLC2A1 axis is essential for FOXD1-mediated PC progression, we inferenced the expression of HOXA11-AS, SLC2A1 or increased the expression of miR-148b-3p in FOXD1-overexpressed cells. Silencing HOXA11-AS, SLC2A1, or enhancing miR-148b-3p markedly reversed the FOXD1-mediated auxo-action of extracellular acidification, oxygen consumption (Fig. 6A, B), and proliferation (Fig. 6C, D), invasion and metastasis (Fig. 6E). Moreover, when PC cells were treated with the glycolysis inhibitor 2-Deoxy-D-glucose (2-DG, 100 mM), FOXD1-mediated auxo-action of proliferation, invasion and metastasis vanished (Supplementary Fig. S4). Thus, we can conclude that both the HOXA11-AS/miR-148b-3p/SLC2A1 axis and aerobic glycolysis are required for FOXD1-mediated PC progression.

### FOXD1 expression is correlated with GLUT1 in PC

**Fig. 7:**
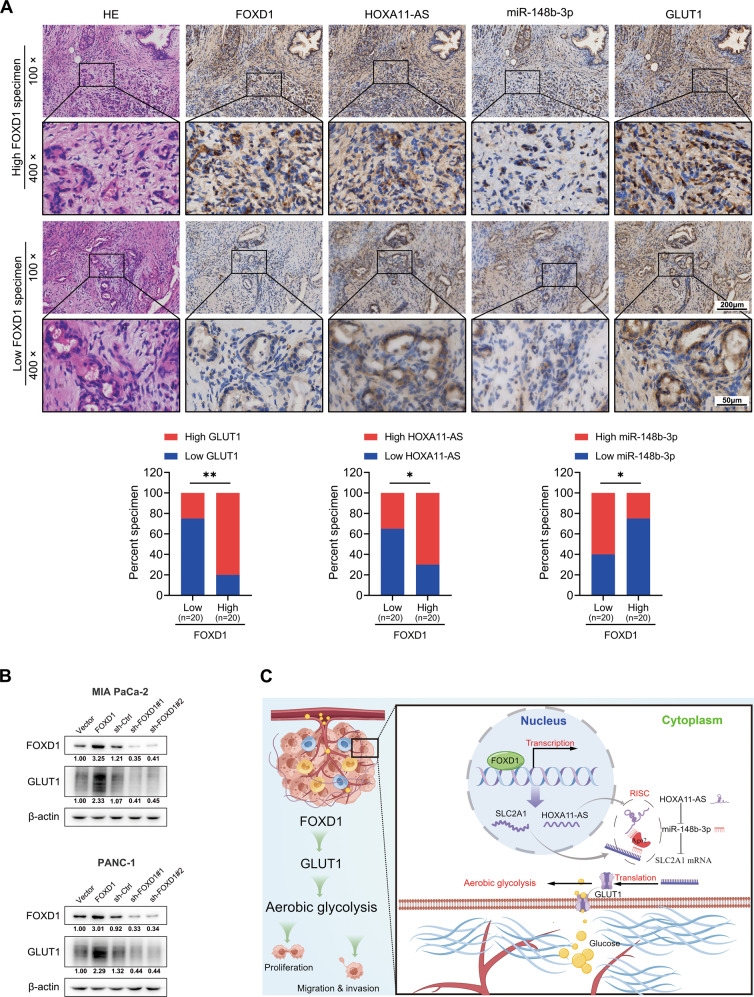
FOXD1 expression correlates with GLUT1 in PC. **A** Representative image of FOXD1, HOXA11-AS, miR-148b-3p, and GLUT1 expression in serial sections of pancreatic cancer specimens detected through IHC and ISH. Pearson’s chi-squared test of the expression of 40 specimens. **B** GLUT1 expression of MIA PaCa-2 and PANC-1 cells with FOXD1 overexpression and knock-down were detected using western blotting. **C** Illustration of how FOXD1 facilitates pancreatic cancer cell proliferation, invasion, and metastasis by regulating GLUT1-mediated aerobic glycolysis, created by Figdraw (http://www.figdraw.com). *p < 0.05; **p < 0.01.

The protein expression of FOXD1 and GLUT1 as well as mRNA expression of HOXA11-AS and miR-148b-3p were measured using IHC or ISH in human PC specimens. As shown in Fig. 7A, relatively high levels of expression of HOXA11-AS and GLUT1 and low expression of miR-148b-3p were detected in FOXD1-rich specimens (Fig. 7A). Consistently, FOXD1 overexpression promoted—and FOXD1 knockdown inhibited—GLUT1 expression in PC cells (Fig. 7B).

## Discussion

PC is a highly lethal disease, with as many fatalities as new cases each year. Even more concerning is the fact that the incidence rate is increasing by 0.5–1.0% each year, PC is expected to become the second leading cause of cancer-related deaths by 2030 [1, 24]. Despite decades of research, the pathogenesis and progression mechanism of PC remains largely unknown, and therapeutic targets to inhibit disease progression are urgently needed [4]. Unlike most other solid tumors, PC tissue contains large amounts of matrix composition, which creates a nutrient-deficient and hypoxic environment for PC cells [25]. As a result, PC cells utilize “metabolic reprogramming,” such as aerobic glycolysis, to meet their excessive energy demand and facilitate malignant behaviors, namely proliferation, invasion, and metastasis [17, 18, 26, 27]. Therefore, discovering the molecular mechanism of enhanced aerobic glycolysis in PC would have immense clinical value.

FOXD1, a member of the FOX transcription factor family, has been found to play vital roles in multiple malignancies: upregulation of VEGFA, which increases tumor angiogenesis in colorectal cancer [28], upregulation of CYTOR, which promotes epithelial-mesenchymal transition and chemoresistance in oral squamous cell carcinoma [11], and upregulation of CTGF, promoting dedifferentiation and resistance to targeted therapy in melanoma [13]. FOXD1 has also been shown to be associated with osteoarthritis [9], gastric intestinal metaplasia [29], glioma [30], cellular differentiation and other deleterious effects [30, 31]. In our multiple analyses—utilizing public databases as well as assays on tissue samples and cell lines—we confirmed that FOXD1 is highly expressed in PC. This elevated expression was found to be positively correlated with indicators of poor clinical prognosis. In addition, our results confirm that FOXD1 overexpression promotes (and FOXD1 repression inhibits) aerobic glycolysis and the ensuing proliferation, invasion, and metastasis of PC cells, both in vitro and in vivo. Collectively, these results can be viewed as evidence that FOXD1 may be an effective prognostic marker and therapeutic target.

PC cells utilize glucose in the process of aerobic glycolysis, meeting the energy requirements for extensive proliferation, invasion, and, ultimately, metastasis [32]. It is known that GLUT1, the translation product of SLC2A1, is commonly overexpressed in PC cells and serves as a crucial transmembrane protein responsible for the uptake of glucose into the cells through facilitative diffusion [19, 33]. In the present study, we demonstrated that FOXD1, as a transcription factor, can bind to specific promoter sequences of SLC2A1 and HOXA11-AS, thus promoting their transcriptional activity.

HOXA11-AS, a homeobox cluster-embedded antisense lncRNA, has also been reported to participate in the initiation and progression of multiple cancers [34–36]. HOXA11-AS acts as a ceRNA that sponges miR-98-5p, thus regulating the expression of Y-box binding protein-2 in oral squamous cell carcinoma and, consequently, the progression of this disease [37]. HOXA11-AS recruits FOXP2, positively regulates the transcription of Cyclin D2, inhibits apoptosis, and promotes cell cycle progression in nephroblastoma [38]. HOXA11-AS can also increase the methylation level of the HOXA11 promoter, activate the Wnt signaling pathway, and strengthen the stem cell characteristics of hepatocellular carcinoma (HCC), promoting HCC development [39]. Recently, HOXA11-AS was reported to function as ceRNAs associated with 5-FU resistance in colon cancer cells [40]. In our study, we found that when the expression of HOXA11-AS was inhibited by small interfering RNA (siRNA), the expression of SLC2A1 was also significantly reduced. By detecting the subcellular localization of HOXA11-AS in PC cells, we found HOXA11-AS mainly localized in the cytoplasm. Given that the function of lncRNAs is dependent to a large degree on their subcellular localization and that the cytoplasm functions mainly via the ceRNA mechanism, we hypothesized that HOXA11-AS also functions via the ceRNA mechanism [41–43]. We found that HOXA11-AS depletion markedly upregulated the miR-148b-3p level and miR-148b-3p overexpression memorably downregulated the SLC2A1 level in PC cells. We also note that miR-148b-3p overexpression clearly reduced the activity of luciferase when the pmirGLO vector was constructed with 3′UTR of HOXA11-AS or SLC2A1 using the WT fragment of the miR-148b-3p binding site instead of the mutant binding site. Moreover, the anti-Ago2 antibody notably enriched HOXA11-AS and miR-148b-3p, which is further evidence that HOXA11-AS, miR-148b-3p, and SLC2A1 appeared in the same RISC in PC cells. These findings indicate that HOXA11-AS functions as a sponge of miR-148b-3p, regulating SLC2A1 expression in PC cells. Together, these results indicate that HOXA11-AS sponged miR-148b-3p alleviated its abundance in PC cells and thus mitigates the degradation of SLC2A1 mRNA by RISC. Lastly, we report that overexpression of FOXD1 promoted—and knockdown of this gene inhibited—the processes of aerobic glycolysis and proliferation, invasion, and metastasis of PC cells.

Stepwise investigation from rescue experiments demonstrated that decreased expression of HOXA11-AS and SLC2A1 expression or increased expression of miR-148b-3p can partially mitigate the promotion effect of FOXD1 on aerobic glycolysis and the proliferation, invasion, and metastasis of PC cells. Inhibition of glycolysis could also reverse FOXD1’s promoting effect on proliferation, invasion and metastasis of PC cell. Finally, we utilized IHC and ISH assays to detect the relevant indicators in PC specimens; the results showed that FOXD1 expression level was positively correlated with HOXA11-AS and GLUT1 and negatively with miR-148b-3p expression. Meanwhile, overexpression of FOXD1 increased—and knockdown of this gene decreased—the GLUT1 protein expression abundance in PC cells. Overall, the outcomes of our analyses support the hypothesis that the FOXD1/HOXA11-AS/miR-148b-3p/SLC2A1 and FOXD1/SLC2A1 axes may play a crucial role in aerobic glycolysis and subsequent progression of PC.

We can conclude that FOXD1 functions as an oncogene in PC: the results of functional and mechanistic assays all point to the function of FOXD1 as a promoter of aerobic glycolysis and the proliferation, invasion, and metastasis of PC cells via increased SLC2A1 transcription and reduced degradation by RISC. Therefore, targeting FOXD1 is a potential therapeutic strategy for the treatment of PC.

## Materials and methods

### Bioinformatics analysis

The GSE16515 data used in this study were accessed through the GEO2R database (http://www.ncbi.nlm.nih.gov/geo/geo2r); genes that were differentially expressed between PC and normal pancreatic tissues were screened by applying the criteria that adj.P.Val < 0.05 and |*log*_2_FC| > 1. The members of FOX family were acquired from HumanTFDB (http://bioinfo.life.hust.edu.cn/HumanTFDB).

GEPIA (http://gepia.cancer-pku.cn/index.html) was used to integrate the TCGA and GTEx data. Gene set enrichment analysis (GSEA) was performed by reanalyzing the TCGA data.

### Sample collection

Samples of PC tissue and adjacent normal pancreatic tissues (90 pairs) were collected from PC patients who had undergone surgery. The collection and usage of clinical specimens were approved by the Ethics Committee of Guizhou Medical University, and each patient signed a written agreement.

### Immunohistochemistry (IHC)

After fixing, embedding, sliding, and deparaffinizing, tissue sections were blocked with 3% H_2_O_2_ and 5% BSA. Then sections were incubated with anti-FOXD1(Invitrogen, Cat No. PA5-35145), anti-KI67 (Proteintech, Cat No. 27309-1-AP), anti-PCNA (Proteintech, Cat No. 10205-2-AP), and anti-GLUT1(abcam, Cat No. ab115730) antibodies at 4 °C overnight. After washing with PBS, immunohistochemical secondary antibody was applied to the sections for 1 h at room temperature, followed by DAB staining, hematoxylin re-staining, and imaging. The results were evaluated blindly by two independent pathologists.

### RNA extraction and RT-qPCR

Total RNA was isolated with Trizol (Invitrogen, Cat No. 10296010) and quantified with Nano Drop ND1000. The cDNA was then synthesized using the Primescript RT Master Mix Kit (Takara, Cat No. RR047A), and qPCR was carried out with the use of TB Green® Premix Ex Taq™ (Takara, Cat No. RR420A). The relative expression was calculated using the 2^− ΔΔCT^ method, in which β-actin or U6 served as the reference gene for internal control. The corresponding primer sequences are given in Supplementary Tables S1 and S2.

### Western Blot

Cells were harvested and lysed in RIPA buffer (BOSTER, Cat No. AR0105) supplemented with protease and phosphatase inhibitors for 10 min on ice. Then cellular debris were removed by centrifugation at 12,000 rpm for 15 min at 4 °C and protein concentrations were determined by BCA Kit (BOSTER, Cat No.AR0197). 50 μg of total protein were subjected to denaturing 10% SDS-PAGE, and then transferred to a membrane for subsequent blotting with specific antibodies. Anti-FOXD1(Invitrogen, Cat No. PA5-35145), anti-GLUT1(abcam, Cat No. ab115730), anti-β-actin (Proteintech, Cat No.66009-1-Ig). All the full and uncropped western blots are uploaded as ‘Supplemental Material’.

### Cell culture

The hTERT-immortalized acinar-to-ductal intermediary cells isolated from the adult pancreas (HPNE) and six PC cell lines (BxPC-3, Capan-2, HPAF-II, MIA PaCa-2, PANC-1, and SW1990) were obtained from the American Type Culture Collection. HPNE and BXPC-3 cells were maintained in RPMI-1640 medium (Gibco) containing 10% fetal bovine serum (FBS; Gibco), and Capan-2, HPAF-II, PANC-1, MIA-PaCa2, and SW1990 were cultured in DMEM medium (Gibco) containing 10% FBS. All cells were cultured in a humidified atmosphere with 5% CO_2_ at 37 °C. All the cell lines had been authenticated through STR profiling and confirmed to be mycoplasma-free.

### Cell infection and cell transfection

Flag-tagged FOXD1-overexpressing, short hairpin RNAs (sh-RNA) targeting FOXD1, and their corresponding control lentivirus were obtained from Genechem (Shanghai, China). The stable cell lines with FOXD1 overexpression and knockdown were screened through culture in puromycin (0.5 µg/mL) for 7 days. The miR-148b-3p mimics, small interfering RNAs (siRNAs) for HOXA11-AS and SLC2A1 were obtained from RiboBio (Guangzhou, China). The mimics and siRNAs were transfected with Lipo3000 (Invitrogen, Cat No. L3000015) according to the manufacturer’s instructions. For the target sequences of sh-RNAs, si-RNAs and sequences of mimics, see Supplementary Tables S3 and S4.

### Extracellular acidification rate and oxygen consumption rate

One day prior to performing the assay, the cells were plated in XF96 cell culture microplates (Seahorse, Cat No. 101085-004) at a density of 4 × 10^4^ cells/well. The extracellular acidification rate and oxygen consumption rate were then measured using a Seahorse XFe96 Analyzer, according to the manufacturer’s instructions for the Glycolysis Stress Test Kit (Seahorse, Cat No.103020-100) and Cell Mito Stress Test Kit (Cat No.103015-100). Three independent replicates were analyzed.

### EdU staining

The EdU Kit (RiboBio, Cat No. C10310-1) was utilized for the EdU staining process. Cells were plated in 24-well culture plates at a density of 5 × 10^5^ cells/well the day prior to the assay. After incubation with 50 µM EdU for 2 h, the cells were fixed with 4% paraformaldehyde and permeabilized with 0.5% Triton X-100. The cells were then stained with Apollo reaction cocktail and Hoechst according to the manufacturer’s instructions. Three independent replicates were analyzed.

### Colony formation assay

Cells were plated in 6-well culture plates at a density of 1 × 10^3^ cells/well. After conventional culture for 14 days, colonies were fixed with 4% paraformaldehyde and stained with 1% crystal violet. Three independent replicates were analyzed.

### Migration and invasion assay

Transwell chambers (Corning, Cat No. 3422) were used in the migration and invasion assays. In the migration assay, 5 × 10^4^ cells were plated on the upper chambers with 200 µL serum-free medium, and the bottom chambers were filled with 700 µL medium containing 10% FBS. After conventional culture for 24 h, migrated cells were fixed and stained. In the invasion assay, the upper chambers were coated with 25 µL Matrigel (BD, Cat No. 3422356234), and the remaining steps were the same as in the migration assay. Six random microscope views (20 × 10 magnification) per treatment were observed and three independent replicates were analyzed.

### In vivo xenografts and metastasis models

The protocols for the animal experiments was approved by the Ethics Committee of Guizhou Medical University. BALB/cA-nu mice (8-weeks old) were obtained from HFKBio and randomly assigned to each group (n = 5). For the xenograft model, 2 × 10^6^ cells were inoculated in the right flank of the mouse. The volume of the tumor was then measured every 5 days and then excised from the euthanized animal 30 days after injection. For the metastasis model, 1 × 10^6^ cells were injected into the caudal vein of the mouse. Then the lung tissues (same location for each animal) were excised from euthanized animals and prepared for HE staining.

### ChIP

SimpleChIP® Plus Sonication Chromatin IP Kit (Cell Signaling Technology, Cat No. #56383) was utilized for the ChIP assay. A total of 1 × 10^7^ FOXD1 overexpressed PANC-1 cells were fixed with 1% formaldehyde, followed by 0.1 M glycine to stop the reaction. The chromatin was then sheared into fragments of 200–1000 bp in length through ultrasonication, and the fragments were precipitated by using anti-Flag (Cell Signaling Technology, Cat No. #14793) or anti-IgG antibody. Lastly, the co-precipitated DNA was extracted with phenol/chloroform for the ChIP-seq step or amplified through qPCR.

### RNA-seq

RNA-sequencing analyses were performed to compare the three FOXD1 overexpressed PANC-1 cell simples and three control simples. Total RNA of each simple was extracted and RNA-sequencing analyses were performed on the Novaseq 6000 platform. Differentially expressed genes (DEGs) were identified by the criteria that |log_2_FC| > 1 and *p* < 0.05.

### Dual-luciferase reporter assay

The wild-type (WT) or mutant (MT) sequences with binding sites for FOXD1 in HOXA11-AS or SLC2A1 promoter regions were inserted into the pGL4.20 luciferase reporter vector (Promega, Cat No.E675A). Cells were plated in 96-well culture plates at a density of 5 × 10^3^ cells/well for 24 h and transfected with 150 ng luciferase reporter plasmid coupled with 30 ng pGL-4.74 Renilla control plasmid (Promega, Cat No.E692A) as an internal control.

The wild-type (WT) or mutant (MT) sequences with binding sites for miR-148b-3p in HOXA11-AS or SLC2A1 3′UTR were inserted into the pmirGLO vector (Promega, Cat No.E1330). Cells were plated in 96-well culture plates at a density of 5 × 10^3^ cells/well for 24 h and transfected with 100 ng plasmid coupled with 50 nM miR-148b-3p mimics.

Subsequently, the firefly luciferase activity and Renilla luciferase activity were measured, and the firefly luciferase activity was normalized to Renilla luciferase activity after transfection for 48 h. Three independent replicates were analyzed.

### RNA immunoprecipitation

The RNA-Binding Protein Immunoprecipitation Kit (Millipore, Cat No. 17-700) was utilized for the RIP assay. PC cells were lysed with RIP buffer and treated with magnetic beads conjugated to anti-Ago2 or anti-IgG antibody. The immunoprecipitated RNAs were then extracted according to the manufacturer’s instructions and quantified through RT-qPCR. Three independent replicates were analyzed.

### CCK8

After confirmation of infection or transfection effect, cells were plated in 96-well culture plates at a density of 3 × 10^3^ cells/well (six duplicate wells per group). At the indicated time intervals after the conglutination of cells, 10 µL CCK-8 solution (MedChemExpress, Cat No. HY-K0301) was added to each well. The absorbance of each well was measured at 450 nm after conventional culture for 2 h. Three independent replicates were analyzed.

### ISH

The expression of HOXA11-AS and miR-148b-3p in 40 samples of frozen PC tissues was assessed using biotin-labeled HOXA11-AS and miR-148b-3p probes designed by RiboBio. Briefly, the frozen sections (on slides) were digested with 20 µg/mL proteinase K, fixed with 4% paraformaldehyde, and dehydrated with ethanol. The slides were then incubated with the corresponding probes according to the manufacturer’s instructions. Lastly, digoxin substrate was used to visualize HOXA11-AS and miR-148b-3p signals, and hematoxylin was used to stain the nucleus.

### Statistical analysis

The results were analyzed using SPSS 23(IBM) and are presented as mean ± standard deviation. Differences between groups were analyzed using the two-sided unpaired Student’s t-test. Survival was analyzed through Kaplan–Meier curves. The expression relationships between HOXA11-AS, miR-148B-3p, and SLC2A1 were discerned through Pearson correlation analysis. Statistical significance was set at  *p* < 0.05.

## Supplementary information

Supplementary figure legends

Supplementary Fig.S1

Supplementary Fig.S2

Supplementary Fig.S3

Supplementary Fig.S4

Supplementary Tables

Reproducibility checklist

The full length uncropped original western blots

## Data Availability

All data generated or analyzed in this study are included in the published article and supplementary information files. The datasets used and/or analyzed during the current study are available from the corresponding author upon reasonable request.
