# Supplementary material for: FOXD1 facilitates pancreatic cancer cell proliferation, invasion, and metastasis by regulating GLUT1-mediated aerobic glycolysis
Source: Cell Death Dis. 2022 Sep 3;13(9):765. doi: 10.1038/s41419-022-05213-w (PMC9440910; doi:10.1038/s41419-022-05213-w)
Supplement: Supplementary file 1 — Supplementary figure legends [file 41419_2022_5213_MOESM1_ESM.docx]

**Supplementary figure legends**

**Supplementary Fig.S1: OS and RFS survival curves of PC patients with different expression level of FOXC1, FOXF2, FOXL1, FOXM1, FOXP2 and FOXQ1 based on TCGA database.**

**Supplementary Fig.S2: Co-expression analysis of miR-148b-3p with HOXA11-As and SLC2A1 in PC patients based on TCGA** **database.**

**A** Co-expression analysis of miR-148b-3p with HOXA11-As in 178 PC patients based on TCGA database. **B** Co-expression analysis of miR-148b-3p with SLC2A1 in 178 PC patients based on TCGA database.

**Supplementary Fig.S3: The stable FOXD1 overexpression, knockdown and their corresponding control MIA PaCa-2 and PANC-1 cell lines were constructed.**

**A** FOXD1 overexpression and knockdown efficiency were detected by RT-qPCR in MIA Paca-2 and PANC-1 cells. **B** FOXD1 overexpression and knockdown efficiency were detected by Western blot in MIA Paca-2 and PANC-1 cells. **p* < 0.05; ***p* < 0.01.

**Supplementary Fig.S4: Aerobic glycolysis is essential for FOXD1-mediated PC progression.**

**PC cells infected with FOXD1-overexpressing lentivirus were treated with 2-DG. A, B** CCK8 and EdU staining were employed to evaluate the proliferation capacity. **C** Transwell assays were performed to evaluate the invasive and metastatic capacities. **p* < 0.05; ***p* < 0.01.
