# Supplementary material for: FOXD1 facilitates pancreatic cancer cell proliferation, invasion, and metastasis by regulating GLUT1-mediated aerobic glycolysis
Source: Cell Death Dis. 2022 Sep 3;13(9):765. doi: 10.1038/s41419-022-05213-w (PMC9440910; doi:10.1038/s41419-022-05213-w)
Supplement: Supplementary file 6 — Supplementary Tables [file 41419_2022_5213_MOESM6_ESM.docx]

| **Supplementary table S1. Primers sequences of RT-qPCR** | | |
| --- | --- | --- |
| **Gene** | **Primers** | **Sequence( 5′ to 3′)** |
| FOXD1 | Forward primer | TGAGCACTGAGATGTCCGATG |
|  | Reverse primer | CACCACGTCGATGTCTGTTTC |
| HOXA11-AS | Forward primer | TTGCCAATCGGGTCACAGCGG |
|  | Reverse primer | TCCAGTGCTGGTCTTCGTTGA |
| SLC2A1 | Forward primer | GGCCAAGAGTGTGCTAAAGAA |
|  | Reverse primer | ACAGCGTTGATGCCAGACAG |
| β-actin | Forward primer | CATGTACGTTGCTATCCAGGC |
|  | Reverse primer | CTCCTTAATGTCACGCACGAT |
| miR-148a-3p | Forward primer | GCGCGTCAGTGCACTACAGAA |
|  | Reverse primer | AGTGCAGGGTCCGAGGTATT |
| miR-148b-3p | Forward primer | TCAGTGCATCACAGAACTTTGT |
|  | Reverse primer | ACAAAGTTCTGTGATGCACTGA |
| miR-301b-3p | Forward primer | CAGGTGCTCTGACGAGGTTG |
|  | Reverse primer | TGGTCCCAGATGCTTTGACA |
| miR-3666 | Forward primer | ACGAGACGACGACAGAC |
|  | Reverse primer | CAGTGCAAGTGTAGATGCCGA |
| miR-4295 | Forward primer | GGGCAGUGCAAUGUU |
|  | Reverse primer | CAGTGCGTGTCGTGGAGT |
| U6 | Forward primer | CTCGCTTCGGCAGCACA |
|  | Reverse primer | AACGCTTCACGAATTTGCGT |

| **Supplementary table S2 Primer sequences of ChIP-PCR** | | | |
| --- | --- | --- | --- |
| **Promoter** | **Region** | **Primers** | **Sequence( 5′ to 3′)** |
| HOXA11-AS | 1 | Forward primer | GCAGTTGGGCAGAAGGAG |
|  |  | Reverse primer | TCAAAGGCGTCGCTGTTT |
|  | 2 | Forward primer | ACCCGATGTTGAAATTGAC |
|  |  | Reverse primer | GAGAACACCGAGTGACGAT |
|  |  |  |  |
| SLC2A1 | 1 | Forward primer | GGAGAACCAGGGACACTTC |
|  |  | Reverse primer | GCACCAGTTTACACGCTCA |
|  | 2 | Forward primer | AAGGAGGATGAGGTGGTGT |
|  |  | Reverse primer | GTATGGAGCCCTGAAACAG |
|  | 3 | Forward primer | AGGCTAAGGTGGGAGGAT |
|  |  | Reverse primer | GAGGTGCAATTTCCAGATAA |
|  | 4 | Forward primer | GGAGGCGGAGGTTACAGT |
|  |  | Reverse primer | ATCCTGGGCGAGTTCCTG |
|  | 5 | Forward primer | CAGGAACTCGCCCAGGAT |
|  |  | Reverse primer | GCATACCCATCTCAAACC |
|  | 6 | Forward primer | GGAGACAGGGAAGGGAGA |
|  |  | Reverse primer | CCTCGTAGGGTTGTAGAAAGA |
|  | 7 | Forward primer | CGGGGTCCTATAAACGCTACG |
|  |  | Reverse primer | GGACTCCCACTGCGACTCTGA |

| **Supplementary table S3 Sequences of mimics** | |
| --- | --- |
| **Mimics** | **Sequences( 5′ to 3′)** |
| miR-148b-3p | UCAGUGCAUCACAGAACUUUGU |
| Control | UCACAACCUCCUAGAAAGAGUAGA |

| **Supplementary table S4 Target sequence of shRNA and siRNA** | |
| --- | --- |
| **RNA** | **Target sequence( 5′ to 3′)** |
| shFOXD1#1 | TCCAGTGTCGAGAACTTTA |
| shFOXD1#2 | GTATATCGCGCTCATCACT |
| si-HOXA11-AS #1 | CGAAAGCACGUAAUCGCCGGUGUAA |
| si-HOXA11-AS #2 | AGGCCAAGUCCGAGUUCCAUUUCUU |
| si-SLC2A1 | AUCAUCAGCAUUGAAUUCCTT |
